# Supplementary material for: Complexome profiling of the Chlamydomonas psb28 mutant reveals TEF5 as an early PSII assembly factor
Source: Plant Cell. 2025 Mar 17;37(6):koaf055. doi: 10.1093/plcell/koaf055 (PMC12164590; doi:10.1093/plcell/koaf055)
Supplement: koaf055_Supplementary_Data [file koaf055_supplementary_data.zip › psb28_interactive.zip!/plots/intensity/qualityPlots/peptides/NUO9_Cre07.g327400.t1.1.html]

### Cre07.g327400.t1.1

Trivial name: NUO9  
SeqCoverage [median]: 44.30  
Mass: 32.14 kDa  
  
 

| Sequence | MissedCleavage | Mass [Da] | wtIntensity | muIntensity | Score |
| --- | --- | --- | --- | --- | --- |
| DFPMTGYQEVR | 0 | 1341.60 | 2588.57 | 4572.63 | 123.63 |
| DHTNTQFK | 0 | 989.46 | 4528.17 | 1569.87 | 126.67 |
| ETWDMFGVFFSGHPDLR | 0 | 2039.92 | 926.51 | 0.00 | 68.26 |
| FEVVYHLLSPR | 0 | 1358.73 | 16147.78 | 24597.06 | 96.10 |
| ILTDYGFTGHPLR | 0 | 1488.77 | 7148.84 | 3175.06 | 74.46 |
| ILTDYGFTGHPLRK | 1 | 1616.87 | 15240.02 | 20726.11 | 95.42 |
| KTTDKDYIASEHMQAFGNTQLTDYIIK | 2 | 3130.53 | 2582.03 | 0.00 | 76.78 |
| RVVSEPLELTQEFR | 1 | 1701.90 | 3534.45 | 3953.36 | 82.55 |
| TTDKDYIASEHMQAFGNTQLTDYIIK | 1 | 3002.43 | 5627.97 | 0.00 | 60.52 |
| VFNTANWFER | 0 | 1282.61 | 3281.24 | 0.00 | 96.34 |
| VVSEPLELTQEFR | 0 | 1545.80 | 26151.15 | 29585.98 | 234.90 |
| YFDFNSPWETLNR | 0 | 1687.76 | 3080.61 | 0.00 | 199.33 |
|  |  |  | 90837.3 | 88180.1 |  |
